# Family Structure and Subsequent Anxiety Symptoms; Minorities’ Diminished Return

**DOI:** 10.3390/brainsci8060097

**Published:** 2018-05-31

**Authors:** Shervin Assari, Cleopatra Howard Caldwell, Marc A. Zimmerman

**Affiliations:** 1 Department of Psychology, University of California, Los Angeles (UCLA), Los Angeles, CA 90095, USA; 2 Department of Psychiatry, University of Michigan, Ann Arbor, MI 48104, USA; 3 Department of Health Behavior and Health Education, School of Public Health, University of Michigan, Ann Arbor, MI 48104, USA; cleoc@umich.edu (C.H.C.); marcz@umich.edu (M.A.Z.); 4 Center for Research on Ethnicity, Culture, and Health, School of Public Health, University of Michigan, Ann Arbor, MI 48104, USA; 5 Prevention Research Center, School of Public Health, University of Michigan, Ann Arbor, MI 48104, USA

**Keywords:** ethnic health disparities, socioeconomic position, populations, race/ethnicity, Blacks, African Americans, anxiety

## Abstract

**Background:** Minorities’ Diminished Return (MDR) theory suggests that socioeconomic position (SEP) may have a smaller effect on health and well-being of members of the minority than the majority groups. **Aim:** Built on the MDR theory, this study compared Whites and African Americans for the effects of three family SEP indicators (family type, parental education, and parental employment) during adolescence on subsequent symptoms of anxiety 18 years later during young adulthood. **Methods:** Flint Adolescents Study (FAS), 1994–2012, followed 359 youth (ages 13 to 17, 295 African American and 64 Whites) for 18 years. The independent variables were family type, parental education, and parental employment during adolescence. The dependent variable was subsequent symptoms of anxiety, measured using the Brief Symptom Inventory (BSI), 18 years later. Age and gender were the covariates and race/ethnicity was the focal effect modifier (moderator). Four linear regression models were estimated to investigate the effects of the three family SEP indicators at age 15 on subsequent symptoms of anxiety at age 33 in the pooled sample and also by race/ethnicity. **Results:** In the pooled sample, having married parents at age 15 was inversely associated with symptoms of anxiety at age 33. We found an interaction between race/ethnicity and family type, indicating a smaller protective effect of having married parents against symptoms of anxiety for African American compared to White participants. The other two SEP indicators did not show any effect and did not interact with race/ethnicity on the outcome. **Conclusion:** In support of the MDR theory, marital status of parents during adolescence protects White but not African American young adults against anxiety symptoms. Diminished return of SEP is one of many underlying mechanisms involved in shaping racial and ethnic disparities in anxiety, however, that is often overlooked. Future research that examines economic and social policies and programs that can equalize the health gains that follow SEP resources among racial groups would be a useful next step.

## 1. Introduction

A large body of empirical and theoretical work has documented the strong protective effects of socioeconomic position (SEP) on a wide range of physical and mental health outcomes [1,2,3,4,5,6,7]. High SEP measured by education [8], income [1,4,5], employment [9,10], and marital status [11,12,13,14] reduces risk of morbidity [15] and mortality [16,17,18]. SEP indicators such as education, income, and marital status also protect populations against poor mental health outcomes such as anxiety and depression [14,19].

Demographic factors such as race/ethnicity have been shown to alter how people gain health benefits from their resources [20,21,22,23]. According to the Minorities’ Diminished Return (MDR) theory [24,25], equal SEP indicators generate unequal health gains for the majority and minority group members [24,25,26]. Researchers have provided extensive support for the MDR theory by showing a smaller protective effect of SEP indicators for the health of racial minorities such as African Americans compared to Whites [22,24,25,27]. Educational attainment [21], income [28], employment status [29], and marital status [30] have all shown stronger health effects for the socially privileged (dominant) than the socially disadvantaged (historically oppressed) group. This theory suggests that racial/ethnic minority status restricts the health gain that usually follows SEP resources [31,32], suggesting that race/ethnicity and SEP have multiplicative rather than additive effects on racial disparities [24,25]. The MDR theory argues that economic and health return of SEP indicators are smaller for non-Whites than Whites because of structural racism [24,25] and interpersonal discrimination [33]. As a result, eliminating racial differences in SEP does not result in a full elimination of racial and ethnic health disparities, and such a goal requires addressing racial and ethnic disparities in gains from SEP as well as disparities in access to SEP resources [24,25]. 

Although research has established a link between high SEP and better mental health [19], less is known about diminished return of SEP indicators on anxiety than depression [20,23,34,35,36] and suicide [37]. For African Americans, high SEP may operate as a risk factor for clinical and subclinical depression [20,23,34]; however, very few studies, if any, have investigated these patterns for anxiety.

### Aims

The current study compared African American and White youth for the effect of family SEP (family type, parental education, and parental employment) during adolescence on subsequent symptoms of anxiety during young adulthood 18 years later.

## 2. Materials and Methods

### 2.1. Design and Setting

The Flint Adolescent Study (FAS) is an 18-year longitudinal study of youth in Flint, Michigan. This prospective study started in 1995 (wave 1) and ended in 2012 (wave 12) [38].

### 2.2. FAS Design and Methods

The FAS followed a high-risk group of youth that were susceptible to school dropout and substance use for up to 18 years. This study followed youth regardless of remaining in school, as well as those who experienced school dropout. 

### 2.3. FAS Participants and Sampling

Participants were enrolled from four local public high schools in Flint Community Schools. Non-random sampling was used to draw participants. The study participants were all ninth graders at entry to study (average age 14.5). Students were enrolled in the fall semester of their ninth grade if their grade point average (GPA) was 3.0 or less in eighth grade and if they did not have a known diagnosis of a developmental/emotional impairment. Although most participants were from working-class households, only 1 out of 4 families included married biological parents. The study had a very low refusal rate. The study participants represented 92% of the eligible youth in the Flint public high schools.

### 2.4. Analytical Sample for Current Analysis

Data for the current analysis were limited to the wave 1 (year 1995) and wave 12 (year 2012) of the participants who were a part of the study during the 18 years of follow up. The retention rate was 90% from wave 1 to wave 4; 75% from wave 4 to wave 8, and 65% from wave 9 to wave 12. The attrition did not differ between the African American and White youth. The analytical sample of the current study was composed of 359 youth (ages 13 to 17, 295 African American and 64 Whites) who were followed 18 years.

### 2.5. Process and Measures

Most of the data were collected via face-to-face interview, followed by a brief paper-and-pencil survey. The paper-and-pencil survey was considered for collecting data on sensitive items. Interviews lasted 60 min on average. Participants were interviewed annually from 1994–1997, 1999–2003, and 2008–2012, with a total number of 12 times. For the years 1994 to 1997, structured/face-to-face interviews were used for data collection. For the years 2003 to 2008, interviews were conducted in community setting or by telephone. Most interviewers were trained community members or college students who were native to the area. We did not find any effects of interviewer race/ethnicity or gender as a source of bias [38]. 

### 2.6. Measures

#### 2.6.1. Dependent Variable

*Symptoms of Anxiety.* Symptoms of anxiety were measured by the Brief Symptom Inventory (BSI) [39]. The BSI uses six items to assess the frequency of feeling uncomfortable due to anxiety during the past seven days. The root question was “I am now going to read a list of problems and complaints that people sometimes have. During the past week, including today, please tell me how uncomfortable you felt because of the following problems: (1) “Nervousness or shakiness inside”; (2) “Suddenly scared for no reason”; (3) “Feeling fearful”; (4) “Feeling tense or keyed up”; (5) “Spells of terror or panic”; and (6) “Feeling so restless you couldn’t sit still”. Responses were on a Likert scale ranging from 1 (“not at all uncomfortable”) to 5 (“extremely uncomfortable”). All six items were then averaged to calculate the anxiety symptoms, with higher score indicating more symptoms. This scale has acceptable reliability (Cronbach’s alpha is 0.78) [40,41,42].

#### 2.6.2. Independent Variables

Three family SEP (family type, parental education, and parental employment) during adolescence were the independent variables. Family type was reflective of family structure, operationalized as a dichotomous variable (1 married parents vs. 0 not married family). Other family SEP indicators included employment status of parents (parents employed, parents unemployed) and highest education level of parents. While employment status of parents was treated as a dichotomous variable, highest education level of parents was operationalized as a continuous measure.

#### 2.6.3. Covariates

Baseline age and gender (0 male, 1 female) were the control variables. Age (years) was treated as a continuous variable, while gender was treated as a categorical variable.

#### 2.6.4. Moderator

Race/ethnicity was the focal effect modifier (moderator). Race/ethnicity was self-identified and was recorded as African American/Black, White, and mixed race/ethnicity (African American and White). As very few individuals were mixed race/ethnicity (*n* = 20), we operationalized race/ethnicity as a dichotomous variable (African American or mixed race/ethnicity 1, Whites 0). In this study, respondents self-identified their race/ethnicity which was conceptualized as a social rather than biological measure. In this study, race/ethnicity is viewed as a salient social construct that shapes how a social group is treated by society. In this view, race/ethnicity is a proxy of access to the opportunity structure, rather than innate fixed abilities.

### 2.7. Analytical Plan

For descriptive purposes, we reported mean and frequency, and standard deviation (SD). For bivariate analysis we used independent sample *t*-test and Pearson Chi square to compare study variables between African Americans and Whites. We also used Spearman correlation test in the pooled sample and by race/ethnicity to estimate the associations between study variables. For multivariable analysis, we used linear regression models. Adjusted b (regression coefficient), 95% confidence intervals (CIs), and *p*-values were reported. 

We ran four linear regression models. In all our models, three family SEP indicators at baseline (age 15) were the independent variables, symptoms of anxiety at wave 12 (age 33) as the dependent variable, and demographic characteristics were the covariates. Race/ethnicity was the focal effect modifier (moderator). The first two linear regression models were fitted to the overall sample. *Model 1* did not include any interaction terms. *Model 2* included three race/ethnicity by SEP indicator interaction terms. Subsequently, we estimated race/ethnicity-specific linear regression models for Whites (*Model 3)* and African Americans (*Model 4*).

## 3. Results

### 3.1. Descriptive Statistics

This study included 359 youth (ages 13 to 17, 295 African American and 64 Whites) who were followed for 18 years. Table 1 summarizes the descriptive information for the pooled sample and by race/ethnicity. At baseline, African Americans were less likely to be from families with an employed parent and married parents in comparison to Whites. African Americans and Whites had comparable age, gender, baseline parental education, and symptoms of anxiety 18 years later (Table 1).

### 3.2. Bivariate Correlations

Table 2 presents the results of three sets of correlation matrix in the pooled sample and by race/ethnicity. In the pooled sample, none of the SEP indicators at baseline were correlated with symptoms of anxiety 18 years later. Marital Status of the family at baseline was correlated with symptoms of anxiety 18 years later for White but not African Americans (Table 2).

### 3.3. Linear Regression Models in the Pooled Sample

Table 3 summarizes the results of two linear regression models in the overall sample with family SEP indicators at baseline (wave 1) as the independent variables, anxiety symptoms at wave 12 as the dependent variable, and demographic factors as covariates. *Model 1* included the main effects but not the interaction terms. *Model 2* included main effects as well as interaction terms between race/ethnicity and SEP indicators. *Model 1* showed that marital status (married parents) at wave 1 was associated with lower symptoms of anxiety at wave 12 net of all study covariates. *Model 2* showed a significant interaction between the effects of race/ethnicity and marital status (married parents) at wave 1, indicating that the effect of having married parents at baseline was smaller for African American compared to White individuals (Table 3).

### 3.4. Linear Regression Models by Race/Ethnicity

Table 4 summarizes the results of two linear regression models that were specifically fitted for Whites (*Model 3*) and African Americans (*Model 4*). *Model 3* in Whites showed an inverse association between being from a family with married parents at baseline and subsequent anxiety symptoms at wave 12. *Model 4* showed that among African Americans, being from a family with married parents at baseline was unrelated to anxiety symptoms at wave 12 (Table 4).

## 4. Discussion

Supporting the MDR theory [24,25], this study documented racial/ethnic variation in the effects of family SEP at age 15 and subsequent symptoms of anxiety at age 33. While being from a family with married parents at baseline was associated with lower anxiety symptoms at wave 12 for Whites, the protective effect of having married parents was absent for African Americans. This is another example that health gain from SEP is unequal for race/ethnic groups, and compared to Whites, African Americans are at a disadvantage. As these patterns were not found for two of three SEP indicators, the results were only suggestive. 

Although this is not the first study to provide support for MDR theory, it is one of the first studies that shows that this theory also holds for symptoms of anxiety. Most of the literature on this theory has focused on physical rather than mental health outcomes [43,44,45,46,47]. From studies on mental health outcomes, most of the research has focused on depression and suicide [34,37,48], and less is known about anxiety.

Our finding extends MDR theory to a new outcome, namely anxiety [24,25]. Previous research has well documented smaller health effects of educational attainment, income, and employment, and physical health outcomes such as chronic medical conditions, obesity, and mortality are also well documented [21,22,26,28,29]. Similar patterns have also been shown for health risk behaviors such as substance use [22,49]. In several studies, psychological assets such as self-efficacy, affect, and coping have shown stronger effects on Whites’ than African Americans’ health status [50,51,52,53,54,55,56,57,58,59,60]. Family SES generates more income for White than African American families [61,62].

The MDR theory argues that structural racism [24,25] and discrimination [33] reduce the economic return of SEP indicators for non-Whites. Due to the existing racism in American society, Whites consistently maintain higher benefits and gains than African Americans. Many social welfare policies that benefit minorities are regarded to be un-American by the dominant group, which also has the political power. Tax cuts still benefit the wealthy more than the middle class, and Whites and African Americans receive education of different quality. Whites and African Americans still live separately, given the existing residential segregation [24,25,29].

The MDR theory does not blame African Americans and other non-White groups for having a tendency to mismanage or waste their economic resources. Instead, the theory attributes the diminished returns to the very fabric of American social structure that fails African Americans and other minorities at each SEP. In US, non-White groups with high ambitions and aspirations pay extra psychological costs (minorities’ tax) for their social mobility. This extra cost takes its toll by placing high-SEP African Americans at an increased risk of poor mental health [20,36,37,63].

In contrast to existing theoretical frameworks such as Multiple Disadvantage [64] and Double [34,65], Triple [66], and Multiple [67] Jeopardy, the MDR theory suggests that it is Whites, not the minority populations, whose health is more strongly dependent upon SEP resources [67]. The models listed above, however, have traditionally conceptualized minority status as a vulnerable status. In addition, most theoretical models that are being used in the health disparities research attributes the racial gap in health to differential exposures rather than to effects and vulnerabilities [24,25]. Unfortunately, differential effects of SEP indicators across social groups are traditionally overlooked [21,22]. These findings question the assumption that SEP is a universal protective factor, suggesting that health disparity researchers should systemically explore interactions between the health effects of race/ethnicity and SEP resources [24,25]. Given the similarities in the two groups in our data, we are very confident in the results that are due to diminished returns.

We did not find significant interaction between race and parental education and employment. The parental employment measure had limited variance, as most families were lower-middle/working class. This is also the case for parental education which did not have large variance. There are only a few Whites with low education and unemployment. Low statistical power is likely to be the reason for lack of interaction between the other SEP indicators.

### 4.1. Implications

Our findings may have implications for policy and public health practice. Policies and programs should go beyond equalizing access of racial groups to SEP by minimizing the diminished returns of minorities as a strategy to reduce or eliminate racial and ethnic health disparities [24,25]. Tailored programs that address these gaps may be needed to ensure that all social groups equally gain from similar programs, and well-intentioned policies and programs reach populations regardless of race/ethnicity and color. Addressing health disparities should go beyond addressing the racial gap in SEP and reduce the racial variation in the cost of upward social mobility, as well as the prevalent barriers in their daily lives that limit non-Whites’ ability to translate their resources to gain [24,25].

Researchers should consider the diminished health return of SEP as a major source of racial health disparities in the US [68,69,70,71]. Policies that universally increase overall access to SEP resources may unintentionally widen the existing health disparities, given that Whites gain more than non-Whites form the very same resources that become available. Policy and program evaluators should be aware that race/ethnicity may alter the real effect of their interventions and Whites may gain more than non-whites from the very same policy or program. We need to know more about how a policy and program changes the life and health of population sub-groups, without assuming that all groups are ready to take advantage of a new program/policy.

### 4.2. Limitations

Our study had a few limitations. Despite the longitudinal design, the current study was an observational study. The current results should not be interpreted as causal associations between SEP and mental health. As SEP and mental health have bidirectional associations, future research should explore reverse causality between poor mental health and SEP. Another limitation of our study was omission of several covariates. We did not include covariates such as parental mental health, mental health history, and other risk factors of future anxiety. Similarly, all the study variables were limited to individual characteristics. Higher-level characteristics such as community SEP, density of crime and violence in the neighborhood, and composition of racial groups in the neighborhood may all effect discrimination which may explain risk of anxiety for Whites and African Americans. The sample size was not balanced between Whites and African Americans, and the model was under-powered for Whites. Finally, differential validity of anxiety symptoms by race/ethnicity may be a source of measurement bias [72]. Another limitation is combining individuals of mixed ethnicity as African Americans. This may cause a problem, as some studies have found significant differences in the mental health among adolescents/young people who self-identify as being mixed when compared to African Americans [73]. None of the above limitations that affect the current study are fatal flaws. Thus, the study still makes a unique contribution to the field of health disparities.

## 5. Conclusions

To conclude, in the US, race/ethnicity alters the protective effects of family structure during adolescence on subsequent symptoms of anxiety 18 years later. The effect of race/ethnicity is not just through changing the distribution of SEP resources, but also changing their health effects. Health disparities are due to multiplicative not additive effects of race/ethnicity and SEP.

## Figures and Tables

**Table 1:** Descriptive statistics in the pooled sample and by race/ethnicity.

Characteristics	All		Whites		African Americans	
	*n*	%	*n*	%	*n*	%
Gender						
Men	156	43.45	28	43.75	128	43.39
Women	203	56.55	36	56.25	167	56.61
SEP Marital Status *^,a^						
Not Married	260	73.65	32	50.79	228	78.62
Married	93	26.35	31	49.21	62	21.38
SEP Parental Employment *^,a^						
Parents unemployed	78	21.79	8	12.50	70	23.81
At least one parent employed	280	78.21	56	87.50	224	76.19
	**Mean**	**SD**	**Mean**	**SD**	**Mean**	**SD**
Age (years)	14.50	0.62	14.45	0.62	14.50	0.62
SEP Parental Education (Highest Parental Education)	4.26	1.49	4.06	1.49	4.31	1.49
Symptoms of Anxiety ^b^	1.50	0.65	1.53	0.64	1.49	0.65

Notes: SEP, Socioeconomic position, * *p* < 0.05 for comparing African Americans and Whites; ^a^ Pearson Chi square; ^b^ Independent samples *t* student test. 95% CI: 95% confidence interval.

**Table 2:** Correlations in the pooled sample and by race/ethnicity.

Characteristics	1	2	3	4	5	6	7
**All**							
1 Race/ethnicity (African Americans)	1	0.00	0.03	0.07	−0.11 *	−0.24 **	−0.02
2 Gender (Females)		1	−0.03	−0.09	0.03	−0.05	0.15 **
3 Age			1	−0.17 **	−0.09	−0.15 **	0.05
4 SEP Parental Education (Highest Parental Education)				1	0.14 *	0.05	−0.07
5 SEP Parental Employment (At least one parent employed)					1	0.15 **	−0.04
6 SEP Marital Status (Married)						1	−0.09
7 Anxiety Symptoms							1
**Whites**							
2 Gender (Females)	-	1	−0.02	−0.26 *	0.14	0.11	0.14
3 Age			1	−0.01	0.13	0.09	0.15
4 SEP Parental Education (Highest Parental Education)				1	0.18	−0.03	−0.01
5 SEP Parental Employment (At least one parent employed)					1	0.09	0.05
6 SEP Marital Status (Married)						1	−0.32 *
7 Anxiety Symptoms							1
**African Americans**							
2 Gender (Females)	-	1	−0.04	−0.04	0.01	−0.09	0.15 **
3 Age			1	−0.21 **	−0.13 *	−0.21 **	0.03
4 SEP Parental Education (Highest Parental Education)				1	0.14 *	0.10	−0.08
5 SEP Parental Employment (At least one parent employed)					1	0.14 *	−0.06
6 SEP Marital Status (Married)						1	−0.04
7 Anxiety Symptoms							1

Notes: SEP, Socioeconomic position, * *p* < 0.05, ** *p* < 0.01.

**Table 3:** Summary of linear regressions between marital status (married parents) at wave 1 and symptoms of anxiety at wave 12 in the pooled sample.

Characteristics	*Model 1* Main Effects		*Model 2* *Model 1* + Interactions	
	B	95% CI	B	95% CI
Race/ethnicity (African Americans)	−0.08	−0.27–0.10	0.21 **	0.07–0.35
Age	0.04	−0.08–0.15	0.62	−1.21–2.45
Gender (Females)	0.19 **	0.05–0.33	0.05	−0.07–0.17
SEP Parental Education (Highest Parental Education)	−0.02	−0.06–0.03	−0.08	−0.63–0.47
SEP Parental Employment (At least one parent employed)	−0.01	−0.18–0.16	0.01	−0.09–0.12
SEP Marital Status (Married)	−0.15 *	−0.32–0.00	−0.01	−0.19–0.18
SEP Education × Race/ethnicity	-	-	−0.43 **	−0.76–0.11
SEP Employment × Race/ethnicity	-	-	−0.04	−0.16–0.08
SEP Marital Status × Race/ethnicity	-	-	−0.03	−0.21–0.14
Intercept	0.87	−0.93–2.67	0.40 *	0.02–0.78

Notes: SEP, Socioeconomic position, * *p* < 0.05, ** *p* < 0.01.

**Table 4:** Results of linear regression models between marital status (married parents) at wave 1 and symptoms of anxiety at wave 12 in Whites and African Americans.

Characteristics	*Model 1* Whites		*Model 2* African Americans	
	B	95% CI	B	95% CI
Age	0.18	−0.08–0.43	0.02	−0.12–0.15
Gender (Females)	0.26	−0.07–0.59	0.20 *	0.04–0.36
SEP Parental Education (Highest Parental Education)	0.02	−0.09–0.13	−0.03	−0.08–0.03
SEP Parental Employment (At least one parent employed)	0.06	−0.42–0.55	−0.04	−0.23–0.15
SEP Marital Status (Married)	−0.46 **	−0.77–0.15	−0.05	−0.25–0.16
Intercept	−1.35	−5.09–2.38	1.11	−0.97–3.18

Notes: SEP, Socioeconomic position, * *p* < 0.05, ** *p* < 0.01.
